# Pneumatic Nail Gun Injury to the Cardiac Box: A Case Report of Successful Extraction of a Potentially Dangerous Tool

**DOI:** 10.7759/cureus.106885

**Published:** 2026-04-12

**Authors:** İsmail Tombul, Yunus E Kerimoglu, Elgun Valiyev, Ali C Düzgün

**Affiliations:** 1 Department of Thoracic Surgery, Ankara Training and Research Hospital, Ankara, TUR; 2 Department of Cardiovascular Surgery, Ankara Training and Research Hospital, Ankara, TUR

**Keywords:** cardiac box, foreign body migration, intraoperative fluoroscopy, pneumatic nail gun, thoracic penetrating trauma

## Abstract

Pneumatic nail guns are widely used in industrial and domestic settings and can cause injuries that may appear minor externally but can be potentially life-threatening. While most injuries involve the extremities, penetrations within the cardiac box carry a significant risk of myocardial or great vessel injury and may present with deceptive clinical stability.

A 39-year-old male furniture worker presented to the emergency department after an accidental chest injury with a pneumatic nail gun. Despite a potentially hazardous trajectory through the corpus sterni toward the pericardium, the patient remained hemodynamically stable and asymptomatic. Multimodal imaging was performed to evaluate the extent of the injury. A 2-cm, 23-gauge metallic nail was identified in the paracardiac region. Given the high risk of delayed cardiac complications due to the nail's proximity to the pericardium, surgical removal was planned. The foreign body was successfully extracted via a limited sub-sternal incision under local anesthesia and sedation. Intraoperative fluoroscopy was utilized to ensure precise localization of the small-caliber nail and to minimize iatrogenic trauma during extraction. The patient was discharged uneventfully on the first postoperative day.

Penetrating injuries to the cardiac box require a high index of clinical suspicion and prompt radiological evaluation, regardless of hemodynamic status. This case demonstrates that minimally invasive, image-guided extraction using intraoperative fluoroscopy can be safely and effectively performed for small-caliber foreign bodies located near the pericardium, enabling precise removal while minimizing procedural risk.

## Introduction

Pneumatic nail guns are commonly used in civilian settings and in the construction and woodworking industries due to their accessibility and low cost [1]. While injuries most frequently involve the upper extremities and hands, the high penetrating capability of these devices can also result in serious and potentially life-threatening injuries involving the thorax, abdomen, pelvis, facial bones, and skull [2,3].

Penetrating injuries within the cardiac box, defined as the area bounded by the sternal notch superiorly, the xiphoid process inferiorly, and the midclavicular lines laterally, are associated with a high risk of cardiac injury [4]. The clinical assessment of these injuries presents significant challenges, particularly in hemodynamically stable patients. The seemingly benign external appearance can be misleading, as diagnostic pitfalls such as delayed migration or occult cardiac damage may be overlooked during the initial evaluation. Recognizing these uncertainties is crucial for ensuring timely intervention.

Here, we present a case of a 39-year-old male who sustained an accidental penetrating injury to the corpus sterni caused by a 23-gauge nail from a pneumatic nail gun, emphasizing the importance of multimodal imaging and careful surgical management even in the absence of immediate hemodynamic compromise.

## Case presentation

A 39-year-old male furniture worker presented to the emergency department within 30 minutes of sustaining an accidental injury from a pneumatic nail gun. Upon admission, the patient was asymptomatic and had no known comorbidities. Initial vital signs were stable, with a blood pressure of 120/70 mmHg, a heart rate of 75 beats per minute, and an oxygen saturation of 96% on room air. Following the initial assessment, a point-of-care ultrasound (POCUS) focused assessment with sonography for trauma (FAST) scan was performed, which revealed no pericardial effusion or intraperitoneal fluid.

**Figure 1:**
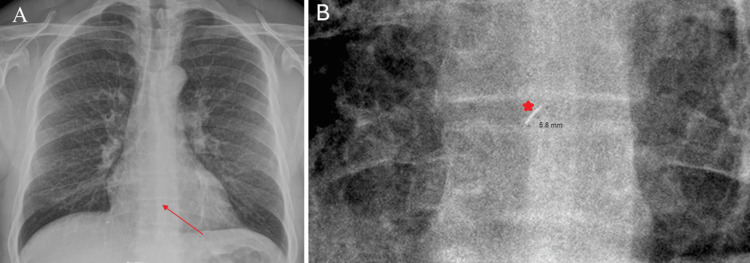
Initial radiological assessment. (A) Posteroanterior (PA) chest X-ray demonstrating a linear radiopaque foreign body (red arrow) superimposed on the lower cardiac silhouette and sternal region. (B) Magnified radiographic view providing a detailed visualization and measurement of the metallic density (red star).

Physical examination revealed a small punctate erythematous entry wound located slightly to the left of the midline at the level of the lower corpus sterni. Diagnostic imaging, including chest X-ray and computed tomography (CT), was completed within two hours of admission. Chest X-ray demonstrated a 5.8 mm radiopaque density superimposed on the cardiac silhouette (Figure 1).

**Figure 2:**
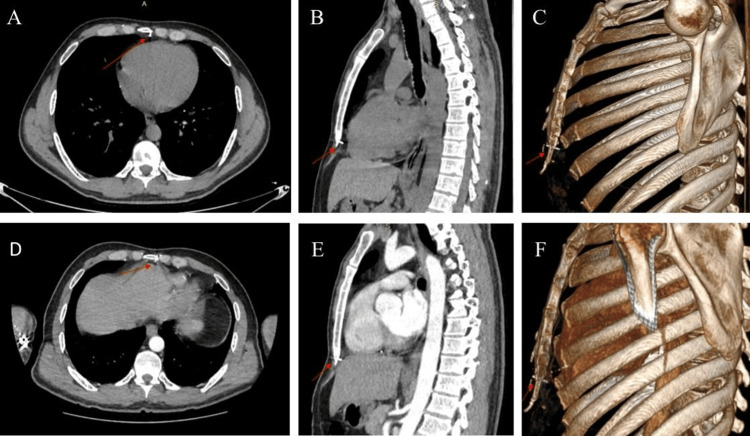
Preoperative thoracic computed tomography evaluation. (A-C) Non-contrast axial, sagittal, and three-dimensional reconstructed computed tomography images demonstrating the metallic nail (red arrows) penetrating the inferior sternum toward the pericardium. (D-F) Contrast-enhanced axial, sagittal, and three-dimensional reconstructed images showing the relationship of the foreign body (red arrows) with cardiac structures. A subtle displacement of the nail is observed between sequences, secondary to respiratory motion.

Non-contrast thoracic CT revealed a 2 cm foreign body traversing the inferior sternum and extending toward the pericardium (Figures 2A-2C). Contrast-enhanced thoracic CT, performed to assess potential cardiac injury, localized the foreign body at the supradiaphragmatic level, terminating within the paracardiac fat tissue. Notably, slight positional changes were observed secondary to respiratory motion (Figures 2D-2F). Transthoracic echocardiography (TTE) revealed no pericardial effusion or signs of cardiac tamponade.

**Figure 3:**
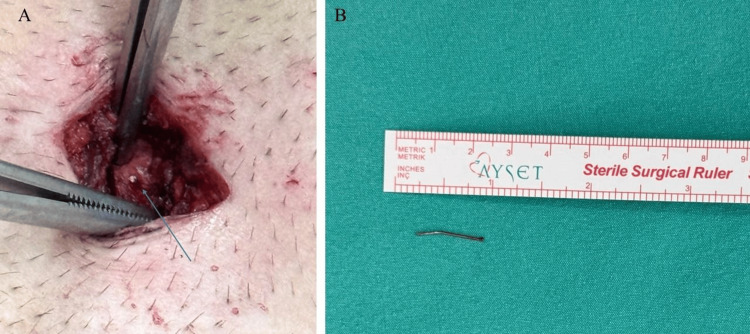
Surgical exploration and extracted foreign body. (A) Intraoperative photograph demonstrating precise localization of the metallic nail (blue arrow) within the substernal space. (B) Macroscopic view of the successfully extracted 23-gauge, 2-cm pneumatic nail, shown alongside a surgical ruler for scale.

Given the high-risk trajectory, the patient was taken to the operating room for surgical intervention within four hours of his initial presentation. Under sedation with intravenous midazolam (2 mg) and fentanyl (50 mcg), and local anesthesia using 10 mL of 2% prilocaine, a 4 cm incision was made at the junction of the lower sternum and the xiphoid process. The foreign body was explored and localized under intraoperative fluoroscopic guidance (Figure 3A), and a 2 cm nail-shaped object was successfully extracted (Figure 3B).

Postoperative TTE showed no pathological findings. The patient was discharged uneventfully on postoperative day one.

## Discussion

Injuries caused by pneumatic nail guns are frequently encountered due to their widespread use in the manufacturing and construction industries. Upper extremity injuries, particularly those involving the hands and fingers, account for approximately 75% of all reported nail-gun-related injuries [5]. Although cardiac nail-gun injuries are relatively rare, they carry a high risk of morbidity and mortality. Vosswinkel et al. reported a mortality rate of 25% in their clinical series [1]. Therefore, diagnostic and therapeutic interventions must be performed promptly, especially in injuries involving the cardiac box region.

The hemodynamic presentation of these patients is often stable at admission. However, when hemodynamic instability occurs, it is most commonly due to cardiac tamponade secondary to pericardial effusion [6]. Current management algorithms recommend that hemodynamically unstable patients undergo immediate surgical intervention without extensive diagnostic workup. Conversely, hemodynamically stable patients should undergo detailed imaging, such as chest X-ray (CXR) or CT, to evaluate potential complications and determine the trajectory of the foreign body [7]. In our case, the patient’s hemodynamic stability allowed for a comprehensive multimodal radiological evaluation, which was crucial for planning the surgical approach.

Rapid and systematic radiological evaluation is mandatory in penetrating thoracic injuries caused by pneumatic nail guns. CXR serves as a useful first-line imaging modality for rapid localization of metallic foreign bodies. However, CT is superior for evaluating lung contusion, hemothorax, and mediastinal structures [8]. Furthermore, bedside TTE plays a crucial role in the emergency setting by enabling rapid detection of pericardial effusion and cardiac tamponade. In our case, multimodal imaging allowed accurate determination of the nail trajectory through the sternum and confirmed the absence of acute cardiac complications prior to surgical intervention.

Surgical intervention is generally recommended in all patients with penetrating nail-gun injuries, regardless of initial hemodynamic status, due to the inherent risk of occult injury and delayed migration. Even in patients who are initially stable without pericardial effusion, retained foreign bodies may displace over time, driven by continuous cardiac and respiratory motion. Such migration can result in late-onset cardiac injury, pericardial tamponade, or sudden hemodynamic compromise. Several reports in the literature have documented cases where chronically migrating foreign bodies caused significant myocardial and pericardial damage [6,9]. Conservative management of retained cardiac foreign bodies has been described in selected asymptomatic cases, particularly when the object is fully embedded and carries a low risk of migration. However, established management criteria suggest that surgical extraction should be prioritized for foreign bodies diagnosed acutely after injury or those with a high potential for migration, infection, or embolization [10]. In our case, despite the patient’s clinical stability, the nail’s trajectory through the sternum, its proximity to the pericardium, and the subtle displacement observed on imaging necessitated immediate surgical removal to prevent potential delayed cardiac complications.

Penetrating thoracic traumas due to pneumatic nail guns have become more frequent due to widespread industrial use. The diversity in nail materials, diameters, and lengths poses significant challenges in determining the optimal therapeutic approach. In our case, the small diameter of the nail made its surgical exploration difficult. Therefore, the procedure was performed under intraoperative fluoroscopic guidance to ensure precise localization and to minimize potential iatrogenic injury during foreign body extraction.

## Conclusions

Nail-gun injuries to the cardiac box may appear clinically silent despite their potentially fatal trajectory. Careful radiological evaluation and timely surgical intervention are therefore crucial. This case highlights the importance of multimodal imaging and the usefulness of intraoperative fluoroscopy in the safe removal of small-caliber, potentially migrating metallic foreign bodies located near the pericardium. Ultimately, the management of cardiac foreign bodies should be individualized. Surgical removal is indicated in symptomatic cases or when there is a risk of migration and complications, while selected stable, asymptomatic cases may be managed conservatively.
